# Endoscopic trans-sphenoidal pituitary surgery does not impact postoperative nasal quality of life

**DOI:** 10.1007/s00405-023-08203-6

**Published:** 2023-08-31

**Authors:** Thomas Weiland, Verena Gellner, Prisca Pondorfer, David Hortobagyi, Emanuel Maitz, Peter Kiss, Andrea Borenich, Eva Z. Reininghaus, Dietmar Thurnher, Peter Valentin Tomazic

**Affiliations:** 1 Department of Otorhinolaryngology-Head and Neck Surgery, Medical University of Graz, Auenbruggerplatz 26, 8036 Graz, Austria; 2 Department of Neurosurgery, Medical University of Graz, Graz, Austria; 3 Institute for Medical Informatics, Statistics and Documentation, Medical University of Graz, Graz, Austria; 4 Department for Psychiatry and Psychotherapy, Medical University of Graz, Graz, Austria

**Keywords:** Anterior skull base, Endoscopic minimally invasive surgery of the skull base, Endoscopic endonasal approach to the pituitary, Quality of life, Patient reported outcome measure

## Abstract

**Purpose:**

The aim of this prospective longitudinal study was to assess both subjective quality of life using questionnaires and objective examination of nasal function with regard to olfaction, nasal air flow and mucociliary clearance in patients after minimally invasive, turbinate-preserving endoscopic transnasal trans-sphenoidal pituitary surgery.

**Methods:**

Patients undergoing endoscopic transnasal pituitary surgery were recruited prospectively and examined during three study visits, preoperatively and 3 and 6 months postoperatively. We examined nasal function using sniffin' sticks test, rhinomanometry, saccharin transit time test, and endoscopic and radiological scores. In addition, the influence on subjective quality of life and mental health was recorded using the Sinonasal-Outcome-Test-20 (SNOT-20) and the Hospital-Anxiety-and-Depression-Scale (HADS).

**Results:**

20 patients undergoing endoscopic pituitary tumor resections were included. No significant changes in olfaction or mucociliary clearance were noted. Nasal air flow showed a tendency to increase in the postoperative course lacking significance. Both the endoscopy and the radiological scores showed a significant deterioration, especially after 3 months, with a trend towards improvement over time. However, neither the SNOT-20 nor the HADS showed significant changes compared to baseline.

**Conclusions:**

Our concept of minimally invasive endoscopic tumor resections on the pituitary gland with preservation of nasal turbinates shows low morbidity for the patient. Despite objectifiable surgery-associated changes in the nose, nasal physiology in terms of smell, airflow and mucociliary clearance can be preserved and the subjective quality of life of our patients remains stable.

## Background

The endoscopic transnasal trans-sphenoidal approach for surgery of pituitary adenomas has gained increasing popularity over the past decades [1, 2]. Numerous scientific papers have dealt not only with the feasibility, safety and success rates of the new surgical technique but also with the significantly reduced morbidity compared to the classic open access route [3–6]. However, even with minimally invasive endoscopic access to the skull base, there is a need to disrupt healthy nasal structures, such as nasal turbinates, nasal mucosa or the posterior septum depending on tumor localization, extent and anatomical situation. Many studies, especially for endoscopic surgery in chronic rhinosinusitis, have shown that morbidity is significantly reduced if the mucous membrane is preserved [7–10]. Nonetheless, many departments still use routine resection of turbinates or preparation of nasoseptal flaps for defect closure during endoscopic surgeries of the anterior skull base [11, 12]. Although, effects of turbinate resection have been controversially discussed, it may lead to nasal dryness, crusting, reduced smell function, impaired frontal sinus drainage and empty nose syndrome, mainly associated with nasal obstruction [13]. According to surgical principles of Messerklinger and Stammberger [14, 15], physiology of the nose is largely preserved at our department in Graz in endoscopic sinus and skull base surgery. Nevertheless, nasal structures are routinely manipulated or even resected to provide a sufficient corridor to the pituitary gland. The sphenoidal sinus is enlarged and a posterior septectomy is performed to allow access from both nasal cavities. In addition, the mucosa is partially resected and the turbinates are relocated. This remodeling can have a negative effect on nasal function and might cause significant subjective discomfort for the patient. In some publications, the patients’ quality of life after such interventions is surveyed using questionnaires [16–19]. On one hand, however, specific negative factors are often missing with these instruments, on the other hand, they are lacking of objective functional parameters in addition to subjective symptoms.

In a prospective longitudinal study, patients undergoing regular endoscopic pituitary surgery at an academic center with more than two decades of experience in multidisciplinary endoscopic skull base surgery were examined pre- and postoperatively with regard to nasal function. On one hand, subjective quality of life was recorded using specific questionnaires, on the other hand, nasal function was objectified using validated examinations. The aim of this study was to evaluate a possible surgery-related reduction in the quality of life of patients and to compare these subjective assessments with objective parameters of the possible change in nasal function.

## Methods

Between June 2020 and September 2021, patients with benign pituitary tumors were enrolled in this prospective longitudinal observational study undergoing transnasal endoscopic skull base surgery at the Medical University of Graz, Department of Otorhinolaryngology, Head and Neck Surgery, in collaboration with the Department of Neurosurgery. The diagnoses were determined on the basis of radiological diagnostics using computed tomography (CT) and magnetic resonance imaging (MRI) as well as endocrinological blood testing. The surgical indications were made in the multidisciplinary skull base board. During three study visits, preoperatively as well as 3 and 6 months postoperatively, the patients were examined with regard to their subjective quality of life on one hand and their objective nasal function using validated examination methods on the other hand. The patient cohort was limited to 20 patients, since the focus was on high-quality acquisition of a large number of different data per patient in a longitudinal design. To increase statistical comparability, a high level of homogeneity within the collective was ensured.

### Subjective quality-of-life assessment

Subjective quality of life with a focus on nasal function was assessed during all study visits using Sinonasal-Outcome-Test-20 validated in German language (SNOT-20 D GAV). Furthermore, in cooperation with the Department of Psychiatry and Psychotherapy, levels of possible anxiety and depression were recorded using another validated questionnaire in German language (Hospital Anxiety and Depression Scale, HADS-D).

### Objective assessment of nasal function

During each visit, nasal endoscopy was performed with special regard to signs of inflammation, crusting and scarring and documented using the Lund–Kennedy endoscopy score[20].

Based on pre- and postoperative imaging (CT and MRI), the degree of inflammation in the paranasal sinus system was also recorded using Lund–Mackay score [21].

To objectify olfactory function, a standardized smell test (Sniffin' Sticks Test, Burkhart Medizintechnik, Wedel, Germany) was carried out on 12 different odors, both separately and combined for each nasal cavity.

A saccharin transit time test [22] was performed to assess the mucociliary clearance time (MCT) of the nasal mucosa. For this purpose, a drop (100 µl) of 40% glucose solution was applied to the right anterior nasal floor and the time until the patients perceived the sweet taste in the pharynx was recorded.

Furthermore, nasal air flow was evaluated using rhinomanometry (Piston Ltd., Budapest, Hungary). For this purpose, a face mask including a flow meter is pressed onto the patient's face and the integrated pressure transducer is plugged into one nostril to measure nasal inspiratory and expiratory resistance and flow at a pressure of 150 Pa, each with and without decongestion of nasal mucosa.

### Clinical parameters

In addition to demographic data, tumor size and location, histological diagnosis and endocrine function were assessed. All parameters were recorded using Redcap data processing program (Research Electronic Data Capture, Vanderbilt University, USA). Furthermore, possible psychiatric pre-existing conditions were documented. Exclusion criteria were determined as follows: patients who previously underwent surgeries on the nose or with a history of chronic rhinosinusitis with or without nasal polyps. Moreover, allergic rhinitis, a known smell dysfunction or treatment with nasal or systemic steroids in the last 4 weeks were defined as exclusion criteria.

### Surgical procedures

All patients underwent a multidisciplinary transnasal endoscopic procedure performed by an ENT surgeon and a neurosurgeon using a four-handed technique. In every case, intraoperative navigation was used to identify critical structures. The sphenoid sinus was accessed trans-septally, with the middle and superior turbinates being gently deflected laterally. The natural ostium of the sphenoid sinus was then identified and the anterior sphenoid wall largely resected protecting the branches of the sphenopalatine artery. In addition, a posterior septectomy without affecting the Vomer was performed and the intersphenoidal septum was resected for the two-nostril-approach. Thereafter, a mucosal window was prepared, the sella was opened and the dura adequately exposed, incised and the tumor removed. For closure, the mucosa was folded back and, in most cases, sealed with fibrin glue. Special attention was paid to a minimally invasive approach, avoiding turbinate resections and the routine preparation of Hadad flaps. In cases of minor cerebrospinal fluid (CSF) leaks, these were sealed with fibrin sealant patches, such as TachoSil^©^ (Takeda, Tokyo, Japan) and autologous fibrin glue. For larger defects, fascia lata and, if necessary, vascularized flaps are routinely used.

The postoperative regimen includes nasal irrigation for patients without a CSF leak from the first postoperative day. The first postoperative check-up and debridement is scheduled after one month, the following examination takes place after 3 months. In patients after a CSF leak, nasal rinsing is only recommended after 1 month postoperatively, when there is no sign of leakage.

### Statistical analysis

Statistical analysis was performed using R 4.2.2 statistical software (R Core Team, 2022). Variables are presented as medians with interquartile ranges and as absolute numbers and percentages. For non-parametric testing, the Wilcoxon signed rank test was used to compare preoperative and postoperative parameters. Statistically significant α level was set at *p* < 0.05.

## Results

The patient collective of this prospective study comprised 20 patients, 11 of them male (55%), 9 females (45%), with a mean age of 54 years ± 14 years. Exclusively pituitary adenomas, the majority being macroadenomas (90%) and two microadenomas (10%), were treated by four different ENT- and five neurosurgeons, all of whom followed a comparable, structured approach. Active hormone production was found in 14 cases (70%), the rest were functionally inactive (30%). 12 tumors showed extrasellar extension (60%), these were graded using the Knosp score showing Knosp-high-grade adenomas (3a, 3b, 4) in 42% (5/12) of cases[23, 24]. In all cases, a minimally invasive approach was used, with none of the cases requiring turbinate resections or vascularized flaps for defect closure. A minor CSF leak occurred in 5 patients (25%), which could be easily closed with absorbable material and one patient had to be revised because of postoperative bleeding (5%). In 12 cases a complete tumor resection could be achieved (60%), while a total resection had to be avoided in the remaining cases, particularly due to the proximity to critical structures. To be more precise, a complete resection had to be dispensed with in two cases due to tumor extension into the cavernous sinus and heavy bleeding. In the remaining cases, a subtotal resection had to be performed mainly because of scarring in the area of the diaphragm with bleeding and/or CSF flow. Of 9 patients with preoperative hyperpituitarism, 7 patients showed a normalized hormonal status during the course. Postoperative hypopituitarism was diagnosed in only 2 patients (10%). In another 2 patients, a pre-existing, yet stable depression was recorded, the rest had no psychiatric illness in the anamnesis. One patient dropped out of the study on his own wish after the first visit, the rest completed all 3 planned study visits, for a total of 58 visits (97%). The details of the patient and tumor characteristics can be seen in Table 1.

**Table 1:** Patient and tumor characteristics

Patient characteristics	*N* = 20
*Age (years)*	
Mean (SD)	54 (14)
*Sex*	
Male	11 (55%)
Female	9 (45%)
*Tumor*	
Pituitary macroadenoma	18 (90%)
Pituitary microadenoma	2 (10%)
Functional (hormone producing) adenomas	14 (70%)
Preoperative hyperpituitarism	9 (45%)
Growth hormone (acromegaly)	5 (25%)
Adrenocorticotropic hormone (cushing)	3 (15%)
Prolactin (prolactinoma)	1 (5%)
Preoperative hypopituitarism	5 (25%)
Reduced cortical/gonadotropic hormones	3 (15%)
Reduced somatotropic/gonadotropic hormones	1 (5%)
Subtotal hypopituitarism	1 (5%)
Non-functional (hormone inactive) adenomas	6 (30%)
Tumor extension	
Sellar	8 (40%)
Extrasellar	12 (60%)
Suprasellar	9 (45%)
Cavernous sinus	4 (20%)
*Knosp Score*	
Grade 1	6/12 (50%)
Grade 2	1/12 (8%)
Grade 3a	3/12 (25%)
Grade 3b	1/12 (8%)
Grade 4	1/12 (8%)
Total resection	12 (60%)
Subtotal resection	8 (40%)
Functional outcome	
Normalized hormone status postoperatively	7/9 (78%)
Postoperative hypopituitarism	2 (10%)
CSF leak intraoperatively	5 (25%)
CSF leak postoperatively	0 (0%)
Revision surgery	1 (5%)
*Psychiatric disorders (depression)*	2 (10%)
*Turbinate resection*	0 (0%)
*Vascularized nasal flap harvesting*	0 (0%)

### Sniffin’ sticks test

**Fig. 1:**
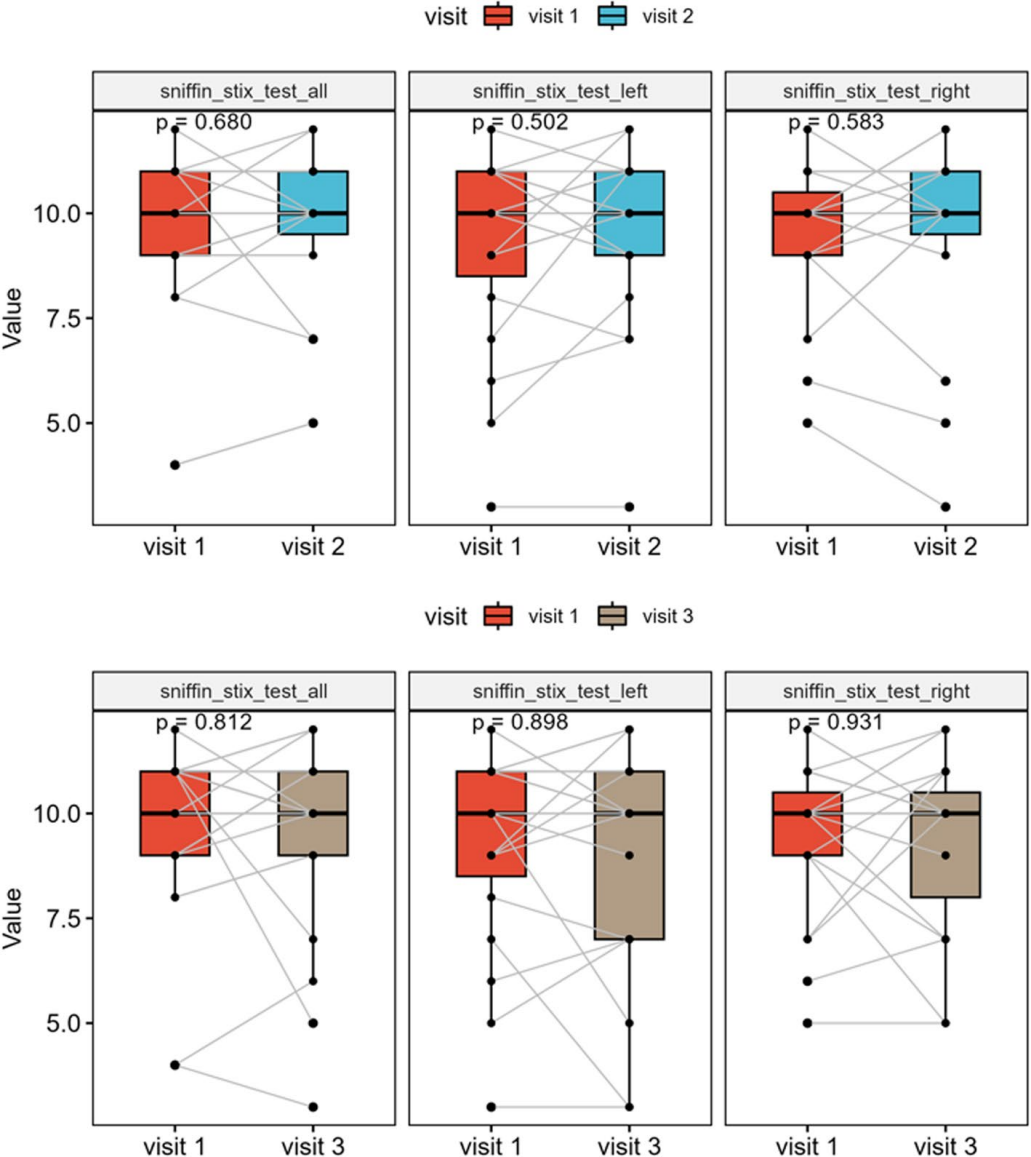
Box plot of the Sniffin’ Stix Test results at 3 and 6 months postoperatively compared to baseline showing no significant change of olfactory function throughout the course (*p* = 0.680 and *p* = 0.812, respectively)

Olfactometry showed no significant differences in either the 3-month or 6-month follow-ups compared to preoperative baseline (*p* = 0.680 and *p* = 0.812, respectively). Median preoperative score for combined testing of both sides was 10 (IQR, 8.75–11) and median postoperative score after 3 months was 10 (IQR, 9.5–11) and after 6 months 10 (IQR, 9–11), respectively (Fig. 1).

### Rhinomanometry

**Fig. 2:**
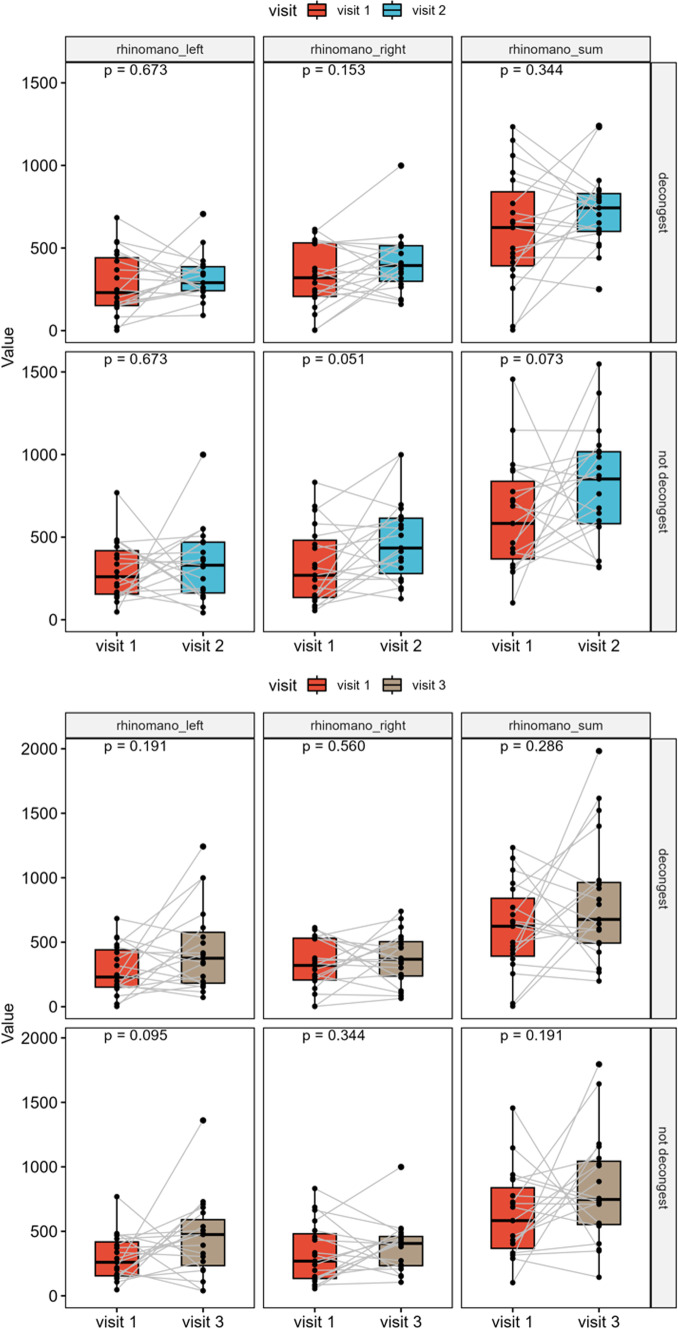
Rhinomanometry results showing no significant change after surgery compared to baseline (without decongestion: *p* = 0.073 and 0.191, respectively; after decongestion: *p* = 0.344 and 0.286, respectively) but a tendency towards improvement of the nasal airflow, especially 6 months postoperatively

Without decongestion, median preoperative score of nasal air flow was 636 (IQR, 386–836) and median postoperative score after 3 months was 852 (IQR, 582–1017) and after 6 months 747 (IQR, 552–1044) (*p* = 0.073 and *p* = 0.191, respectively). After decongestion of the mucosa, median scores were 638 (IQR, 402–805) preoperatively and 743 (IQR, 600–830) after 3 months and 677 (IQR, 494–964) after 6 months, respectively (*p* = 0.344 and *p* = 0.286, respectively) (Fig. 2).

### Saccharin transit time test

MCT was assessed using saccharin transit time test. Median time values (in seconds) were 498 (IQR, 5–769) before surgery, 315 (IQR, 15–576) 3 months postoperatively and 492 (IQR, 218–1090) after 6 months, also showing no significant changes in the pre- to postoperative comparison (*p* = 0.266 and 0.542, respectively).

### Sino-Nasal-Outcome-Test-20 (SNOT-20)

**Fig. 3:**
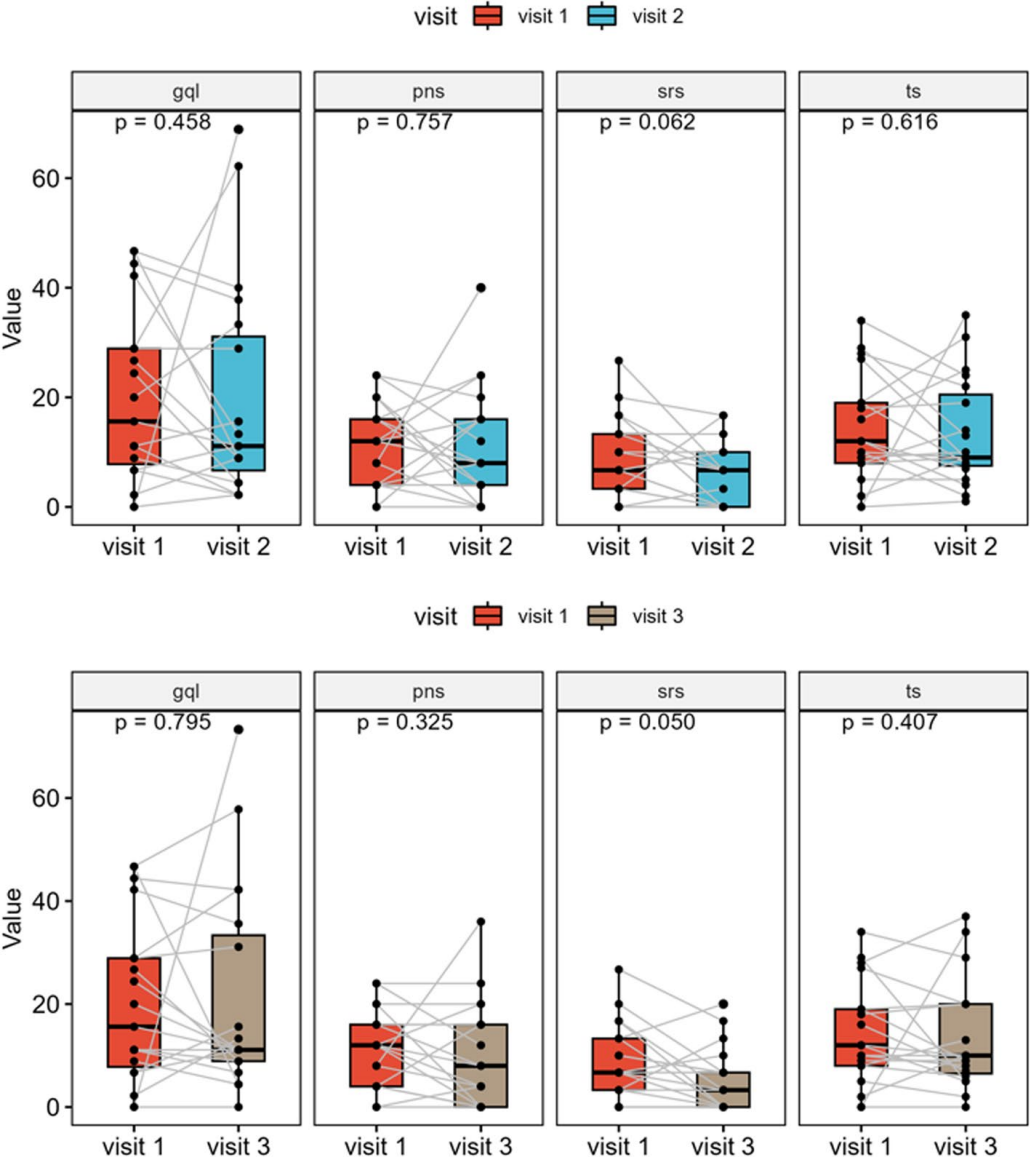
Box plot of the SNOT-20 results showing no significant change in the postoperative course both in the total score (ts: *p* = 0.616 and 0.407, respectively) and in the subcategory “primary nasal symptoms” (pns: *p* = 0.757 and 0.325, respectively), “secondary rhinogenic symptoms” (srs: *p* = 0.062 and 0.050, respectively) and “general quality of life” (gql: *p* = 0.458 and 0.795, respectively)

Median preoperative total score was 11 (IQR, 7–19) and postoperative total scores were 9 (IQR, 8–20) after 3 months and 10 (IQR, 6–20) after 6 months, respectively, showing no significant differences (*p* = 0.616 and *p* = 0.407, respectively). A closer look at the primary and secondary nasal symptom scores revealed similar results before and after surgical therapy. Median primary nasal symptom score was 12 (IQR, 4–16) before surgery, 8 (IQR, 4–16) 3 months postoperatively and 8 (IQR, 0–16) after 6 months, respectively, without significant differences (*p* = 0.757 and *p* = 0.325, respectively). Median secondary nasal symptom scores were 6.7 (IQR, 2.5–13.3) preoperatively, 6.7 (IQR, 0–10) 3 months after surgery and 3.3 (IQR, 0–6.7) during the last study visit, showing no significant differences either (*p* = 0.062 and 0.050, respectively). In addition, with regard to general quality-of-life scores, no significant differences could be determined over the course (*p* = 0.458 and *p* = 0.795, respectively). Median scores were 13 (IQR, 7–29) before surgery, 11 (IQR, 7–31) 3 months after and 11 (IQR, 9–33) 6 months after surgery (Fig. 3).

### Hospital anxiety and depression scale (HADS)

**Fig. 4:**
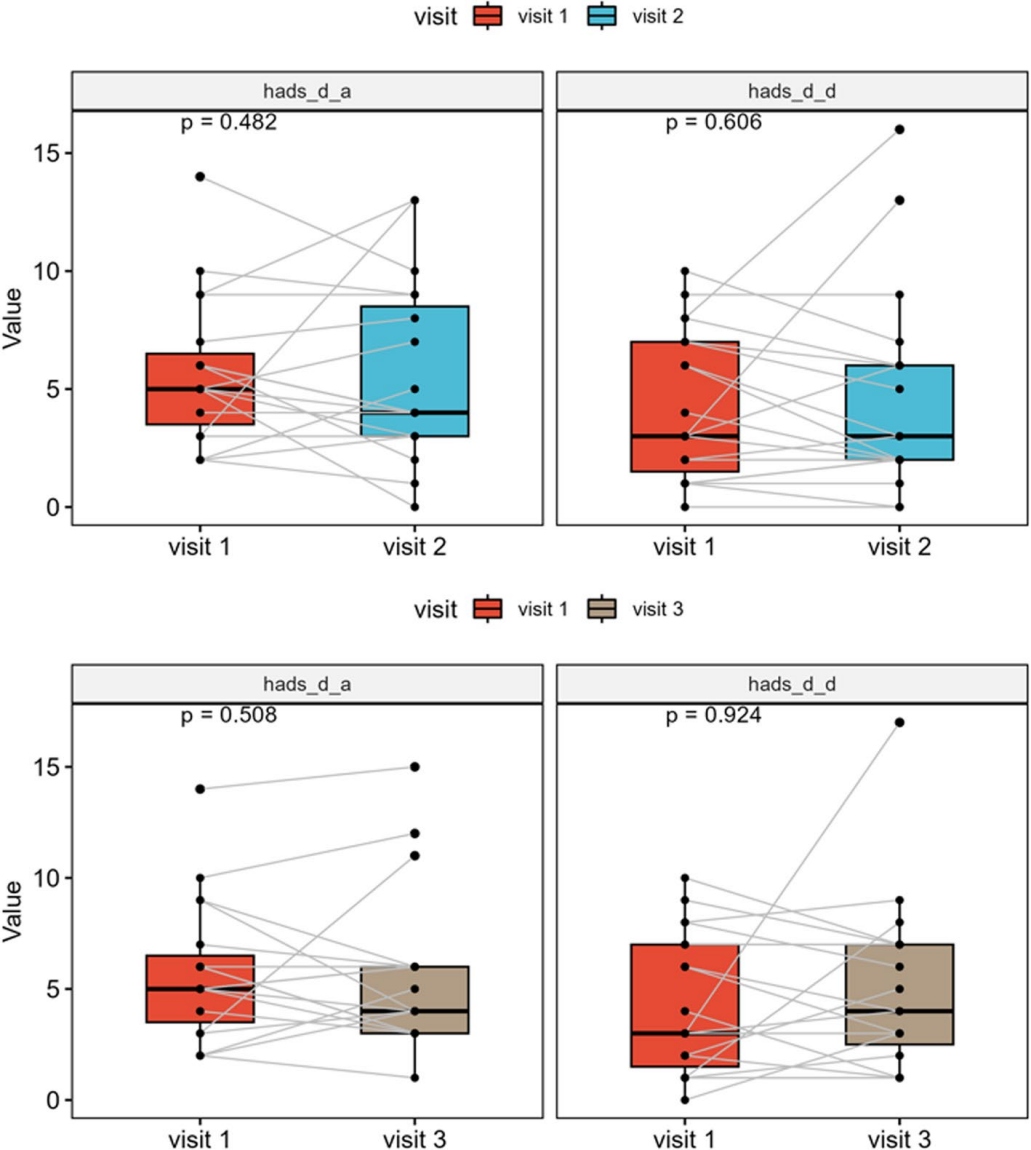
Results of the Hospital Anxiety and Depression Scale revealed comparable values in our patients before and after surgical therapy lacking significant changes (anxiety: *p* = 0.492 and 0.508, respectively; depression: *p* = 0.606 and 0.924, respectively)

When evaluating the HADS results, comparable values were found in the patients before and after surgery (Fig. 4). Median anxiety scores were 5.0 (IQR, 3.8–6.2) preoperatively, 4.0 (IQR, 3.0–8.5) 3 months postoperatively and 4.0 (IQR, 3.0–6.0) 6 months after surgery and did not differ significantly (*p* = 0.492 and *p* = 0.508, respectively). Median preoperative depression score was 3.0 (IQR, 1.75–7), 3.0 (IQR, 2–6) after 3 months and 4.0 (IQR, 2.5–7) 6 months postoperatively, showing also no significant differences (*p* = 0.606 and *p* = 0.924, respectively).

### Lund–Kennedy Endoscopy Score

**Fig. 5:**
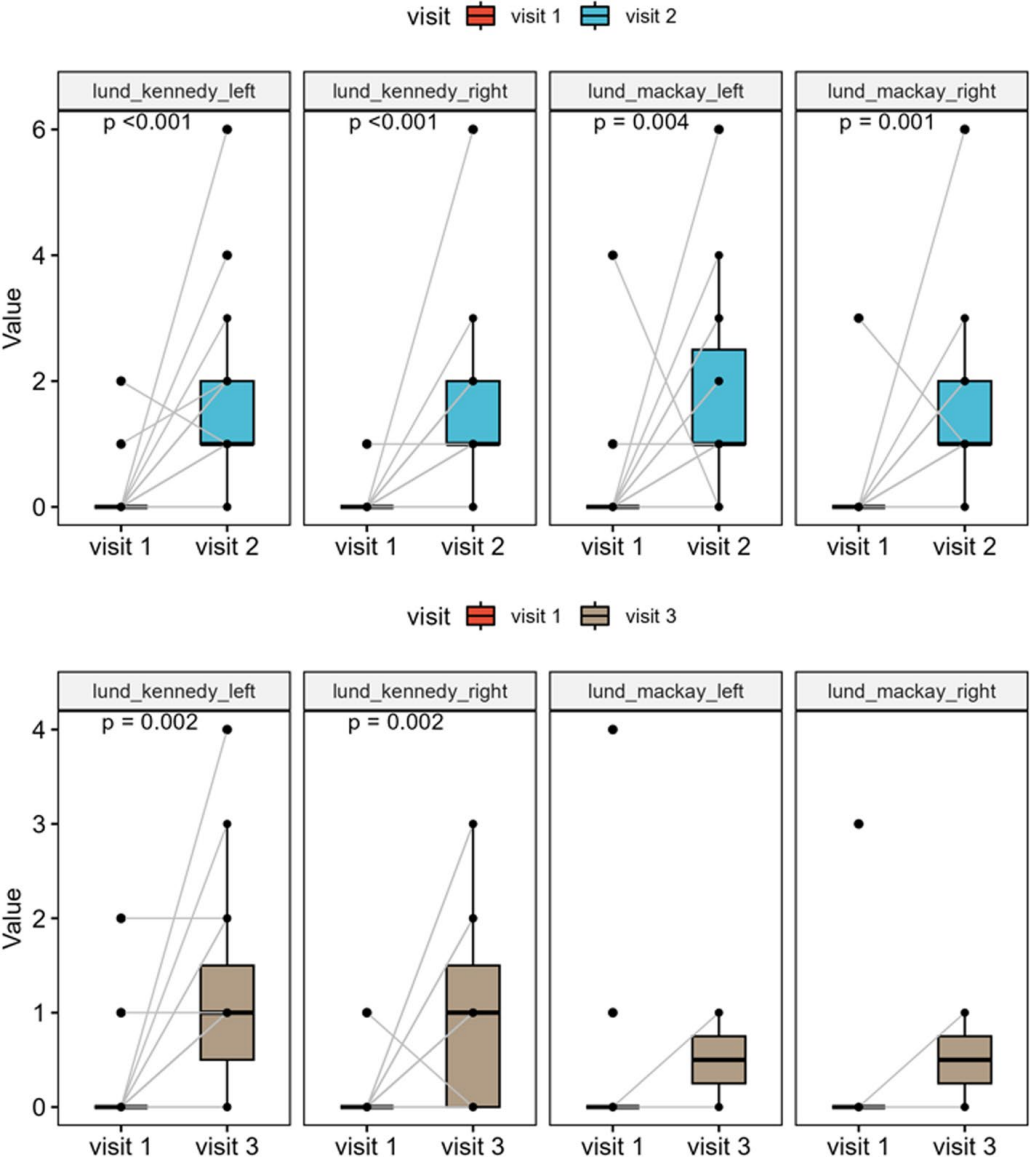
Results of the Lund–Kennedy endoscopy scores show a significant deterioration three and six months after surgery compared to baseline (in both sides *p* < 0.001 and *p* = 0.002, respectively). In particular, a significant rate of synechiae formation (68%) was noted in postoperative endoscopy. Especially additional signs of inflammation such as crusting and secretion led to higher scores during the first postoperative study visit three months postoperatively, which improved over time. A similar picture emerges having a look on the Lund–Mackay scores also showing a significant deterioration (*p* = < 0.01), especially 3 months after surgery, with a trend towards improvement throughout the course

Lund–Kennedy endoscopy scores revealed a significant deterioration after 3 and 6 months compared to the initial findings (in both sides *p* < 0.001 and *p* = 0.002, respectively). Median preoperative score was 0 (IQR, 0–0) for both sides, the median postoperative scores after 3 months were again for both sides 1 (IQR, 1–2) and after 6 months left 1 (IQR, 0.5–1.5) and right 1 (IQR, 0–1.5), respectively. In particular, a significant rate of synechiae formation (68%) was noted in postoperative endoscopy, especially in the area between the middle/upper turbinate and the septum. In addition, there were local signs of inflammation, such as crusting and secretion, particularly during the first postoperative study, which improved over time (Fig. 5).

### Lund–Mackay Score

Radiological scoring showed significant differences in the pre- and 3-month postoperative comparison too (*p* = 0.04 and *p* = 0.001, respectively), in terms of local (partial) shadowing of the paranasal sinus system 3 months after surgery (Fig. 5). Median preoperative scores were 0 (IQR, 0–0) for both sides. Median postoperative scores after 3 months were 1 (IQR, 1–2.5) for the left side and 1 (IQR, 1–2) for the right side and 0.5 (IQR, 0.25–0.75) for both sides after 6 months.

## Discussion

In the present prospective longitudinal clinical study, we examined the influence of endoscopic transnasal trans-sphenoidal pituitary surgery on the patients’ objective nasal function using clinical tests on one hand and patients’ subjective perception of nasal symptoms and their psychosocial impact using questionnaires on the other hand. Following surgical principles of the Messerklinger and Stammberger school, we aim to protect nasal structures, especially nasal turbinates and mucosa, operating as little invasively as possible.

In a previous retrospective study, we recently showed that of 306 endoscopic operations at the anterior skull base, in 91.8% of cases all turbinates could be spared at our department [25]. When examining possible factors influencing the need for turbinate resection, it was shown that this rate was particularly low in pituitary adenomas and significantly higher in larger tumor sizes [25]. In our experience, extended approaches are only indicated in rare extrasellar, particularly far lateral tumor localizations, such as the area of the orbit, to improve visualization and accessibility. Although, there was a relatively high rate of subtotal resections in our study cohort, the analysis showed that the minimally invasive approach was not the limiting factor. In most cases, a complete resection was dispensed due to bleeding and/or CSF flow to avoid risk of injuring critical structures.

In addition to our technique, other minimally invasive surgical approaches have been described so far. Especially, the unilateral (mononostril) transethmoid–paraseptal approach should be mentioned here, in which an ethmoidectomy is additionally performed to be able to lateralize the middle and superior turbinates even further to gain space [26]. This is also described in a four-handed technique and the significant reduction in the risk of postoperative bleeding and a shorter operating time are particularly emphasized by sparing one nasal cavity [27]. Despite having little experience with the unilateral technique, from our point of view; however, the greater maneuverability of the surgeons with the four-handed technique is a great advantage of the bilateral approach. Nonetheless, other groups describe more invasive standard approaches with routine turbinate resections in endoscopic trans-sellar surgery as well, underlining better overview, increased postoperative sinunasal patency and the potential use of free mucosal grafts from the middle turbinate [11, 28–31]. Several studies have shown a significant increase in nasal morbidity in extended approaches with turbinate resection and/or preparation of pedicled mucosal flaps as in malignant tumor resections [32–35]. To investigate how a minimally invasive standard endoscopic approach for trans-sellar tumor resection affects nasal function, we performed pre- and postoperative objective clinical tests recommended in the "European position paper on diagnostic tools in rhinology" in a cohort of pituitary adenoma patients [36]. Neither subjective olfactory perception based on SNOT-20 nor direct olfactometry using the sniffin' sticks test revealed any relevant surgery-related smell disorders in our patients 3 and 6 months postoperatively (Fig. 1). This seems to be in partial agreement with literature findings so far. On a closer look on relevant publications, predominantly subjective olfactory parameters were recorded only using symptom questionnaires, while direct olfactory testing of various odors was neglected, although subjective perception and objective testing sometimes vary. For example, Rioja et al. found in a prospective study that an expanded endoscopic approach and as in another group with a less invasive approach, a subjective smell disorder was recorded using a Visual Analogue Scale (VAS) for at least 3 months after surgery, whereas direct olfactometry using the “Barcelona-Smell-Test-24” revealed no postoperative changes [37]. Bedrosian et al. have noticed a subjective temporary disturbance in sense of smell and taste using the Anterior Skull Base Questionnaire (ASBQ) in their prospective study both after endoscopic surgery on pituitary adenomas and non-pituitary tumors without providing direct olfactometry [38]. Hura et al. reported an even longer-lasting subjective smell and taste reduction after endoscopic skull base surgery using the Anterior Skull Base Nasal Inventory-12 (ASKNI) questionnaire, again without providing direct olfactometry [39]. Hart et al. on the other hand, did not find any differences in olfactory function using the Pennsylvania Smell Identification Test (UPSIT) [40]. Discrepant results are also found when examining whether the use of vascularized flaps influences olfactory function. Using UPSIT, Rotenberg et al. described a smell impairment 6 months postoperatively using vascularized mucosal flaps for reconstruction [12]. Similarly, Kim et al. found a significant odor disturbance up to 6 months using one or two-sided nasoseptal flaps for defect closure based on VAS and direct olfactometry, again with partially diverging results [41]. Tam et al., however, found in their prospective study that both patient groups with and without vascularized septal flaps, suffered from a loss of smell using UPSIT [42]. Sowerby et al. also examined the effect of unilateral middle turbinate resection on smell performance using UPSIT and concluded that there was no significant change [43].

Nasal airflow distortion is also a key symptom for quality-of-life reduction [44]. Using rhinomanometry, significant changes in nasal airflow could be ruled out in our study. The results even show a tendency towards improvement postoperatively, which may be due to improved space conditions of the nasal cavity through turbinate lateralization and posterior septal resection (Fig. 2). In addition, SNOT-20 showed no subjective postoperative nasal obstruction. In this regard, our findings are again consistent with current literature. Schreiber et al. even described a significant postoperative nasal airflow improvement based on rhinomanometry 6 months after surgery [45], while Garzaro et al. found no significant nasal air flow disturbance in 100 patients postoperatively [46]. In those patients with nasal airflow reduction, this was mainly attributed to crusting and synechiae vanishing after appropriate therapy [46, 48].

For assessing MCT of the nasal mucosa, a saccharin transit time test was carried out, again showing no significant differences in the postoperative course. Since no vascularized flap was used for defect closure, and therefore, relevant manipulations on the nasal floor could be avoided, these results correspond to our expectations. Alobid et al. also found no significant change in MCT in patients undergoing a standard surgical approach [35], while the group with an expanded approach including nasoseptal flap reconstruction showed a significant lengthening of MCT [35]. Rioja et al. found mucociliary clearance impairment to persist even up to 1 year after surgery [37].

Lund–Kennedy endoscopy scores deteriorated significantly in the postoperative follow-up. Particularly, secretion, crusting and synechiae formation were responsible. However, during the last study visit, the scores improved again after crusting largely vanished (Fig. 5). Of course, the relevant synechia formation rate of 68% remained, which was mainly observed between the middle/upper turbinate and the septum. In a retrospective study, Schlüter et al. recommended septal splints to reduce postoperative synechiae formation and associated odor and breathing impairment [47]. Since our patients did not subjectively perceive any restrictions despite synechiae, we still see no reason to routinely use prophylactic septum foils. However, we advise using nasal rinses after a certain healing time to clean the nasal mucosa of crusts. Postoperative inflammation and scarring were also reflected in the significant deterioration of Lund–Mackay scores 3 months after surgery (Fig. 5).

Despite significant worsening of endoscopic and radiological scores, the subjective perception of primary and secondary nasal symptoms in SNOT-20 did not deteriorate. In addition, subjective general quality of life showed to be stable over time. HADS was furthermore, used to detect possible symptoms of postoperative anxiety or depression, which also presented to be stable over time (Fig. 4). A review of the available literature shows that quality of life after endoscopic skull base surgery presents to be largely good [18, 19, 34, 48]. In some cases, restrictions are described in the first few months after surgery, mainly related to a reduced smell function which usually improves over time. The degree of surgical invasiveness certainly has an impact on relevant patient symptoms. While operations on malignant tumors are generally linked to quality-of-life impairment, we also find increased morbidity in expanded surgical approaches for benign anterior skull base tumors. Other authors follow the concept of minimal invasiveness and protection of nasal structures as well, which is also reflected in the excellent quality-of-life preservation of their patients [45, 49].

Our study had several limitations such as the lack of specific questionnaires for endoscopic skull base operations, such as the Anterior Skull Base QOL Questionnaire, the Skull Base Inventory (SBI) or the Anterior Skull Base Nasal Inventory (ASKNI-9 and ASKNI-12), which were to the best of our knowledge not validated in German language at the time the study started. This also applies to the successor to the SNOT-20, the SNOT-22. We decided against a self-validation process as this would have gone beyond the scope of the study.

Concerning the limited number of patients, the focus was placed on a manageable, homogeneous, representative group of patients, in which a large number of data should be collected in a longitudinal, high-quality manner to evaluate our minimally invasive standard of care for our endoscopic pituitary procedures.

## Conclusions

The results of our study show that the proven surgical concept of our minimally invasive endoscopic standard approach for operations on the pituitary gland causes low morbidity without relevant objective and subject impairment of nasal function. Despite significantly increased endoscopic and radiological scores postoperatively, in particular due to crusting and synechiae formation, the patient's subjective perception is not negatively affected. Nevertheless, it is essential to inform the patient about the possible occurrence of these inflammatory and scarring processes.

## Data Availability

All data generated or analysed during this study are included in this published article.
